# Mindfulness, Resilience, and Burnout Subtypes in Primary Care Physicians: The Possible Mediating Role of Positive and Negative Affect

**DOI:** 10.3389/fpsyg.2015.01895

**Published:** 2015-12-17

**Authors:** Jesús Montero-Marin, Mattie Tops, Rick Manzanera, Marcelo M. Piva Demarzo, Melchor Álvarez de Mon, Javier García-Campayo

**Affiliations:** ^1^Faculty of Health and Sport Sciences, University of ZaragozaHuesca, Spain; ^2^Miguel Servet University Hospital, University of ZaragozaZaragoza, Spain; ^3^Department of Clinical Psychology, VU University AmsterdamAmsterdam, Netherlands; ^4^Primary Care and Mental HealthLondon, UK; ^5^Department of Preventive Medicine, Mente Aberta – Brazilian Center for Mindfulness and Health Promotion, Universidade Federal de São PauloSao Paulo, Brazil; ^6^Department of Internal Medicine, University of AlcaláMadrid, Spain; ^7^Primary Care Prevention and Health Promotion Research Network (RedIAPP)Zaragoza, Spain

**Keywords:** burnout subtypes, mindfulness, resilience, affect, structural equation modeling, primary care

## Abstract

**Purpose:** Primary care health professionals suffer from high levels of burnout. The aim of the present study was to evaluate the associations of mindfulness and resilience with the features of the burnout types (overload, lack of development, neglect) in primary care physicians, taking into account the potential mediating role of negative and positive affect.

**Methods:** A cross-sectional design was used. Six hundred and twenty-two Spanish primary care physicians were recruited from an online survey. The Mindful Attention Awareness Scale (MAAS), Connor-Davidson Resilience Scale (CD-RISC), Positive and Negative Affect Schedule (PANAS), and Burnout Clinical Subtype Questionnaire (BCSQ-12) questionnaires were administered. Polychoric correlation matrices were calculated. The unweighted least squares (ULS) method was used for developing structural equation modeling.

**Results:** Mindfulness and resilience presented moderately high associations (φ = 0.46). Links were found between mindfulness and overload (γ = −0.25); resilience and neglect (γ = −0.44); mindfulness and resilience, and negative affect (γ = −0.30 and γ = −0.35, respectively); resilience and positive affect (γ = 0.70); negative affect and overload (β = 0.36); positive affect and lack of development (β = −0.16). The links between the burnout types reached high and positive values between overload and lack of development (β = 0.64), and lack of development and neglect (β = 0.52). The model was a very good fit to the data (GFI = 0.96; AGFI = 0.96; RMSR = 0.06; NFI = 0.95; RFI = 0.95; PRATIO = 0.96).

**Conclusions:** Interventions addressing both mindfulness and resilience can influence burnout subtypes, but their impact may occur in different ways, potentially mediated by positive and negative affect. Both sorts of trainings could constitute possible tools against burnout; however, while mindfulness seems a suitable intervention for preventing its initial stages, resilience may be more effective for treating its advanced stages.

## Introduction

Job-related chronic distress is an occupational hazard for healthcare professionals affecting around 38% of primary care personnel, and has been linked to burnout, low health status levels, and outcomes such as worse patient safety and poorer quality of care (Krasner et al., 2009; Al-Sareai et al., 2013; Dolan et al., 2015). This syndrome reflects a situation where there is a lack of harmony between an employee and his/her workplace (Farber, 2000a). It is a psychosocial disorder caused by stressful working conditions, and is a response by workers to a process of maladaptation to chronic distress. Burnout develops progressively as a result of the use by workers of ineffective coping strategies in their attempts to protect themselves from the work-related distress caused by relationships with clients and/or the organization (Maslach et al., 2001). International large-scale studies found that 22% of physicians met criteria for burnout (Linzer et al., 2001). This syndrome negatively affects physicians' self-reported attitudes (such as communication with the patient, empathy, or perceived reciprocity in the patient-physician relationship), and behaviors (such as propensity for medical errors or quality of care delivered; Linzer et al., 2001; Thomas et al., 2007; Williams et al., 2007).

Burnout has been traditionally defined by the dimensions of exhaustion, cynicism, and inefficacy (Maslach et al., 1996). Exhaustion is the feeling of not being able to offer any more of oneself at work, as the consequence of a prolonged exposure to excessive demands. Cynicism is a detached attitude to tasks, colleagues, and recipients of service. Inefficacy is the feeling of not performing tasks adequately and of being incompetent. Recently, a more comprehensive definition of burnout has been proposed to differentiate three different profiles or subtypes (Farber, 2000a). The frenetic subtype is characterized by overload and the perception of jeopardizing one's health to pursue worthwhile results, and is highly associated with exhaustion. The underchallenged subtype is characterized by lack of development, defined as the perception of a lack of personal growth, together with the desire for a more rewarding occupation that better corresponds to one's abilities, and is most strongly associated with cynicism. The worn-out subtype is characterized by neglect, defined as an inattentive and careless response to responsibilities, and is closely associated with inefficacy (Montero-Marín et al., 2011, 2012).

The burnout subtypes can be ordered according to level of dedication to tasks, which affects the way individuals manage their feelings of distress. Altering the level of dedication to tasks may be a way for individuals to exert some control over the balance between efforts and rewards (Farber, 2000b). The frenetic subtype, with its active coping style, is the most engaged profile, while the least dedicated is the worn-out subtype, because of its passive coping style. This classification criterion is consistent with the idea of a developmental transition between the different burnout profiles driven by changes in dedication, from more to less dedicated. Each stage of burnout may correspond to a different pattern of perceived stress as a result of differing levels of dedication (Montero-Marín et al., 2009, 2011, 2012, 2014a).

Mindfulness is a complex phenomenon that can be conceptualized in different ways (Langer, 1989; Demarzo, 2015): as a mental trait or state, characterized by a particular kind of awareness without judgment; as meditation-based practices and programs; and as a socio-cognitive phenomenon (an opposite state of mind, in relation to mindlessness). We have approached mindfulness as a psychological trait that can be assessed by questionnaires, and that influences the health of human beings. In this sense, mindfulness refers to an awareness that emerges by intentionally paying attention to the present experience in a nonjudgmental or nonevaluative way. This particular quality of awareness has been associated with several indicators of physical and psychological health, and can be developed using specific techniques (mindfulness practices) commonly delivered in mindfulness-based interventions (MBIs; Demarzo et al., 2014). MBIs might not only lead to the maintenance of a healthy mental state in health workers, but also to a better quality of care for patients (Watanabe et al., 2015).

Resilience has been characterized as a dynamic and flexible process of adaptation to life changes that could serve as a protective factor against psychological distress and mental disorders. It is the amount of personal strength, energy, and motivation that enables an individual to cope with and recover from stress, and to flourish when faced with adversity (Rutter, 1985; Norris et al., 2008). Resilience is not only important to improve the mental health of health personnel, but also to buffer and minimize the negative consequences of the occupational stress to which they are at risk, with its most adverse result being signs of burnout (Arrogante, 2014).

Personal characteristics such as negative affectivity and disengagement coping are closely related to burnout (Lue et al., 2010; David and Quintão, 2012). In fact, burnout comprises negative perceptions, affect, and behaviors toward work, toward the people who relate with the individual in his/her workplace, and toward his/her actual professional role (Ahola et al., 2010). The improvement of emotion regulation might play a mediating role between mindfulness and resilience, and burnout development by enhancing coping processes (Hoge et al., 2013).

There are few studies assessing potential relationships between mindfulness, resilience, positive and negative affect, and burnout in healthcare professionals or other populations, despite the expected causal paths linking them, and these emphasize the need for new research in this field (Sears and Kraus, 2009; Kemper et al., 2015). The main aim of the present study, and what this study adds to previous research, was to explore the associations of mindfulness and resilience with burnout subtypes, taking into account the possible mediating role of positive and negative affect in primary care physicians.

**Figure 1:**
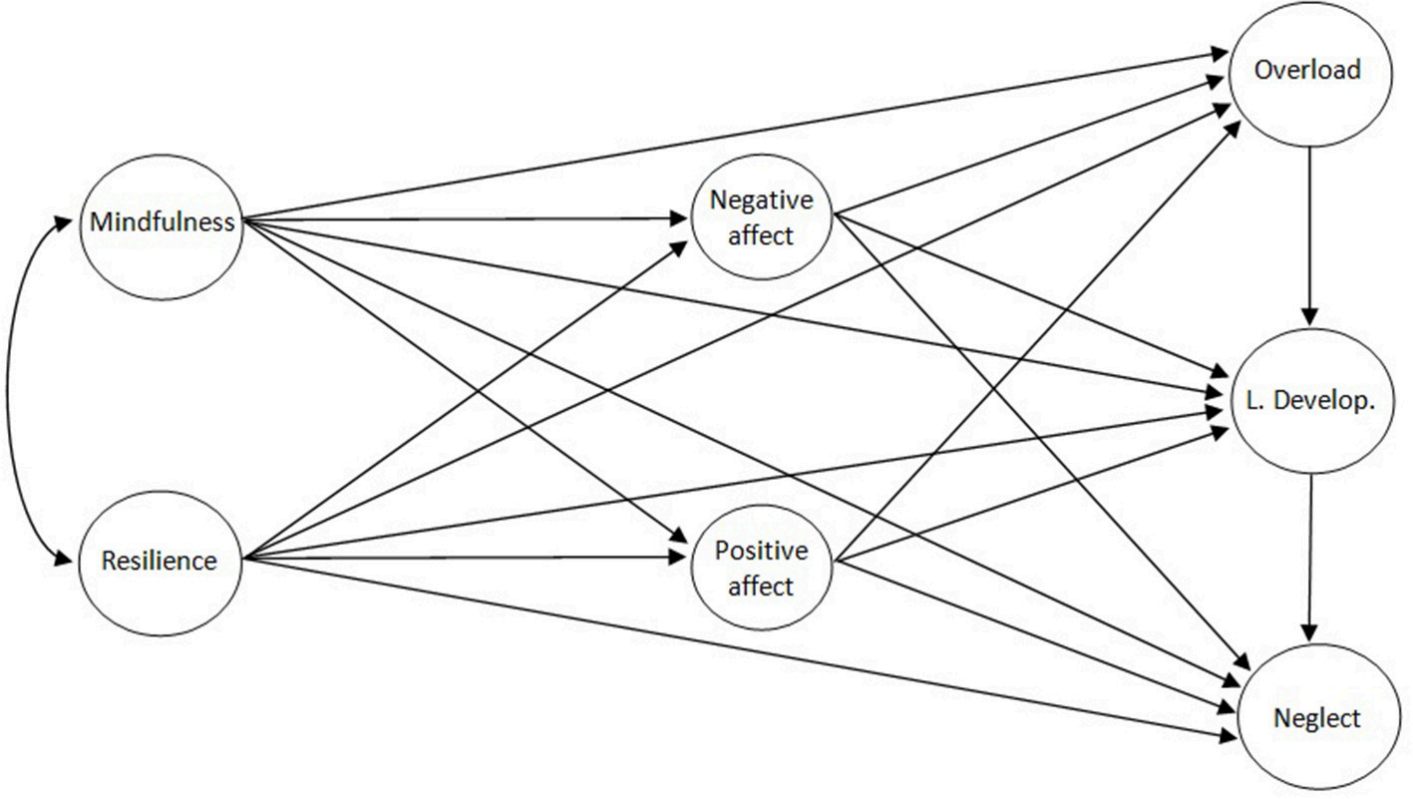
**Hypothetical saturated model**.

We started with the following assumptions: Mindfulness and resilience are strongly related (hypothesis 1); mindfulness and resilience are directly related to the burnout subtypes (overload, lack of development, and neglect), and indirectly through the intermediary latent factors of affect (positive and negative), with different patterns for each burnout profile (hypothesis 2). It was expected that lack of mindfulness would relate most to overload, and lack of resilience to neglect. We also expected important links between the burnout profiles, routed from lowest to highest level of dedication (Hypothesis 3). The saturated hypothetical structural model is shown in Figure 1.

## Methods

### Design

The correlation method was used, with a cross-sectional, individual differences design for data collection. The measurements were obtained by a self-reported online questionnaire (there is no intervention in this study).

### Sample, setting, and ethics

Participants were recruited from a mindfulness course for Spanish primary care physicians, offered by the University of Alcalá de Henares (UAH). The sample size was calculated according to the recommended 10:1 ratio for the number of subjects to the number of test items (Kline, 1998). The study was conducted in April, 2013. An email was sent from the official UAH website to all of the course participants before its commencement, explaining the aims of the research, the voluntary nature of participation, potential benefits and risks, and data confidentiality. This message contained a link to complete the online questionnaire before the start of the course, after providing informed consent. The protocol used was approved through the Ethical Committee of the UAH, Spain.

### Measures

#### Sociodemographic characteristics

Background information collected from the participants included age, gender, marital status, cohabitation, employment status, type of employment contract, and practice of mindfulness.

#### Mindful attention awareness scale (MAAS)

The MAAS (Brown and Ryan, 2003) is a 15-item measure of mindfulness. Each item is rated on a Likert scale between 1 (almost always) and 6 (almost never), in relation to the respondent's everyday experience (e.g., “I rush through activities without being really attentive to them”). The item ratings are averaged to form a total score. Higher scores reflect higher levels of dispositional mindfulness. The scale has been validated in Spanish with appropriate psychometric features (Soler et al., 2012).

#### Connor-Davidson Resilience Scale (10-item CD-RISC)

The CD-RISC (Campbell-Sills and Stein, 2007; Notario-Pacheco et al., 2011) is a 10-item measure of resilience. Each item is rated on a Likert scale from 0 (not at all) to 4 (almost always). The final scores are obtained by summing the response to each of the items (e.g., “I can deal with whatever comes my way”), with higher values indicating higher levels of resilience. The validity and internal consistency are adequate and positively related to sleep quality and mental health (Campbell-Sills and Stein, 2007; Notario-Pacheco et al., 2011).

#### Positive and negative affect schedule (PANAS)

The PANAS (Watson et al., 1988) is a self-report instrument that measure positive and negative affect. This questionnaire consists of a list of 20 adjectives, 10 per subscale (e.g., positive: “interested”; e.g., negative: “guilty”), rated on a 5-point scale, and using the time instructions established by the researcher. Present moment instructions were used in this study. This questionnaire has been validated in Spanish with good psychometric properties (Sandín et al., 1999).

#### Burnout clinical subtype questionnaire (BCSQ-12)

The BCSQ-12(Montero-Marín et al., 2011) is a 12-item measure of burnout subtypes, through the dimensions of overload (e.g., “I overlook my own needs to fulfill work demands”), lack of development (e.g., “My work doesn't offer me opportunities to develop my abilities”), and neglect (e.g., “I give up in response to difficulties in my work”). Participants had to indicate the degree to which they agreed with each of the statements presented according to a Likert scale scored from 1 (totally disagree) to 7 (totally agree). This questionnaire presents good psychometric characteristics (Montero-Marín et al., 2011, 2012).

### Data analysis

Analyses were performed using the SPSS-19.0, FACTOR-9.02, and AMOS-7.0 statistical packages. Mardia's coefficients were evaluated in order to decide the kind of matrix to be used for structural equation modeling (SEM). Polychoric correlation matrices (Olsson, 1979) with regard to the MAAS, CD-RISC, PANAS, and BCSQ items were calculated because of the item distributions (Muthen and Kaplan, 1992). We verified the adequacy of the polychoric correlation matrices, and assessed the determinant, Kaiser-Meyer-Olkin (KMO) index, and Barlett's test of sphericity (Mardia, 1974).

The unweighted least squares (ULS) method was used for developing covariance structures (Jöreskog, 1977). ULS does not provide inferential procedures for assessing model data fit based on the χ^2^ distribution (and therefore, it does not supply significance values for the coefficients), but it does not require any distributional assumptions; it is quite robust and usually converges because of its efficiency in terms of computation. Moreover, in complex solutions it tends to provide less biased estimates of the true parameter values than classical methods; it is an appropriate choice for moderately sized samples; it shows good performance when working with polychoric matrices; it tends to provide accurate estimates even with large models; and it seems to provide better estimates than more complex procedures (Knol and Berger, 1991; Parry and McArdle, 1991; Briggs and MacCallum, 2003; Lee et al., 2012).

We applied ULS from polychoric correlation matrices to test the fit of the measurement models by confirmatory factor analysis (CFA). Lastly, we used structural equations SEM to evaluate the empirical links between the MAAS, CD-RISC, PANAS, and BCSQ-12. In order to evaluate the model fit to the data, we examined the gamma goodness-of-fit index (GFI), the adjusted goodness-of-fit index (AGFI), the root mean square of the standardized residuals (RMSR), the normed fit index (NFI), Bollen's relative fit index (RFI), and the parsimony ratio (PRATIO). GFI and AGFI refer to explained variance, and values >0.90 are considered acceptable (Byrne, 2009). SRMR is the standardized difference between the observed and the predicted covariance, indicating a good fit for values < 0.08 (Hu and Bentler, 1999). NFI measures the proportional reduction in the adjustment function when going from null to the proposed model, and is considered acceptable when >0.90 (Lévy et al., 2006). RFI takes into account the discrepancy for the model evaluated and for the baseline model, and is very good close to 1 (Bollen, 1986). PRATIO is an overall measure of how parsimonious the model is, and it shows how much more efficient the model is than the independence model (Byrne, 2009).

All of these indices are valid for the ULS procedure. Taken together, they provide a reliable evaluation of the solution and additional information regarding absolute, incremental, and parsimonious model-data fit assessment. The factor weights, explained variance, and the association between latent factors—all of which were standardized—were taken into account to examine the pattern of relationships. The unstandardized values of residual errors for the endogenous latent variables in terms of disturbance were also assessed. The initial saturated hypothetical model (Figure 1) considered mindfulness and resilience as independent variables, positive and negative affect as intermediary latent variables, and burnout subtypes as dependent variables. The parsimony of the hypothetical model was improved by dismissal of those standardized path coefficients with very small effects (absolute values < 0.10), using PRATIO in a joint, iterative, and exploratory way (Lévy and González, 2006; Byrne, 2009).

## Results

### Characteristics of participants

A total of 636 primary care physicians from all of the Spanish regions were invited to participate. Of these, 14 (2.2%) did not complete the questionnaire. Therefore, the final sample size was *n* = 622. It comprised adults of European ethnicity between the ages of 28 and 63 years (Mean = 49.32; *SD* = 7.06), 79.9% of whom were women. The main characteristics of participants are shown in Table 1.

**Table 1:** **Characteristics of the participants (*n* = 622)**.

Age, Md (SD)	49.32 (7.06)
Sex, females (%)	497 (79.9)
Stable relationship, yes (%)	463 (74.4)
Residence (%)	
Parents	31 (5.0)
Alone	90 (14.4)
Partner	415 (66.8)
Partner and children	86 (13.8)
Employment (%)	
Employed	606 (97.5)
Unemployed	12 (1.9)
Sick leave	4 (0.6)
Type of contract (%)	
Temporary	98 (15.7)
Permanent	91 (14.7)
Permanent public sector	433 (69.6)
Practice mindfulness, no (%)	595 (95.6)

*Md, Mean; SD, Standard Deviation; Number and percentage (%)*.

### Measurement models

As shown in Table 2, Mardia's multivariate kurtosis coefficients advised the estimate of polychoric correlation matrices in all of the questionnaires. Polychoric matrices showed very good KMO indices (≥0.86), determinant values (≤ 0.006), and Bartlett's statistics (*p* < 0.001), which revealed adequate properties with which to perform CFA. All of the measurement models presented very good fit indices without using correlations between the error terms (GFI ≥ 0.97; AGFI ≥ 0.97; RMSR ≤ 0.06; NFI ≥ 0.96; RFI ≥ 0.96), which meant that the utilization of the instruments was legitimate.

**Table 2:** **Characteristics of the measurement models**.

**Scale**	***M***	**KMO**	**Det**	**Bartlett (df)**	**GFI**	**AGFI**	**RMSR**	**NFI**	**RFI**
*MAAS*	305.79^*^	0.95	< 0.001	4594.60 (105)^*^	0.99	0.99	0.05	0.99	0.98
*CD-RISC*	153.14^*^	0.94	0.006	3093.80 (45)^*^	0.99	0.99	0.04	0.99	0.99
*PANAS*	545.11^*^	0.89	< 0.001	5529.50 (190)^*^	0.97	0.97	0.06	0.96	0.96
*BCSQ-12*	255.74^*^	0.86	0.003	3421.70 (66)^*^	0.99	0.98	0.06	0.98	0.97

M (df), Mardia's multivariate kurtosis coefficient; ^*^p < 0.001; KMO, Kaiser-Meyer-Olkin; Det, Determinant; Bartlet (df), Bartlett's statistic (degrees of freedom); GFI, gamma goodness-of-fit index; AGFI, adjusted goodness-of-fit index; RMSR, root mean square of the standardized residuals; NFI, normed fit index; RFI, Bollen's relative fit index.

### Structural model

**Figure 2:**
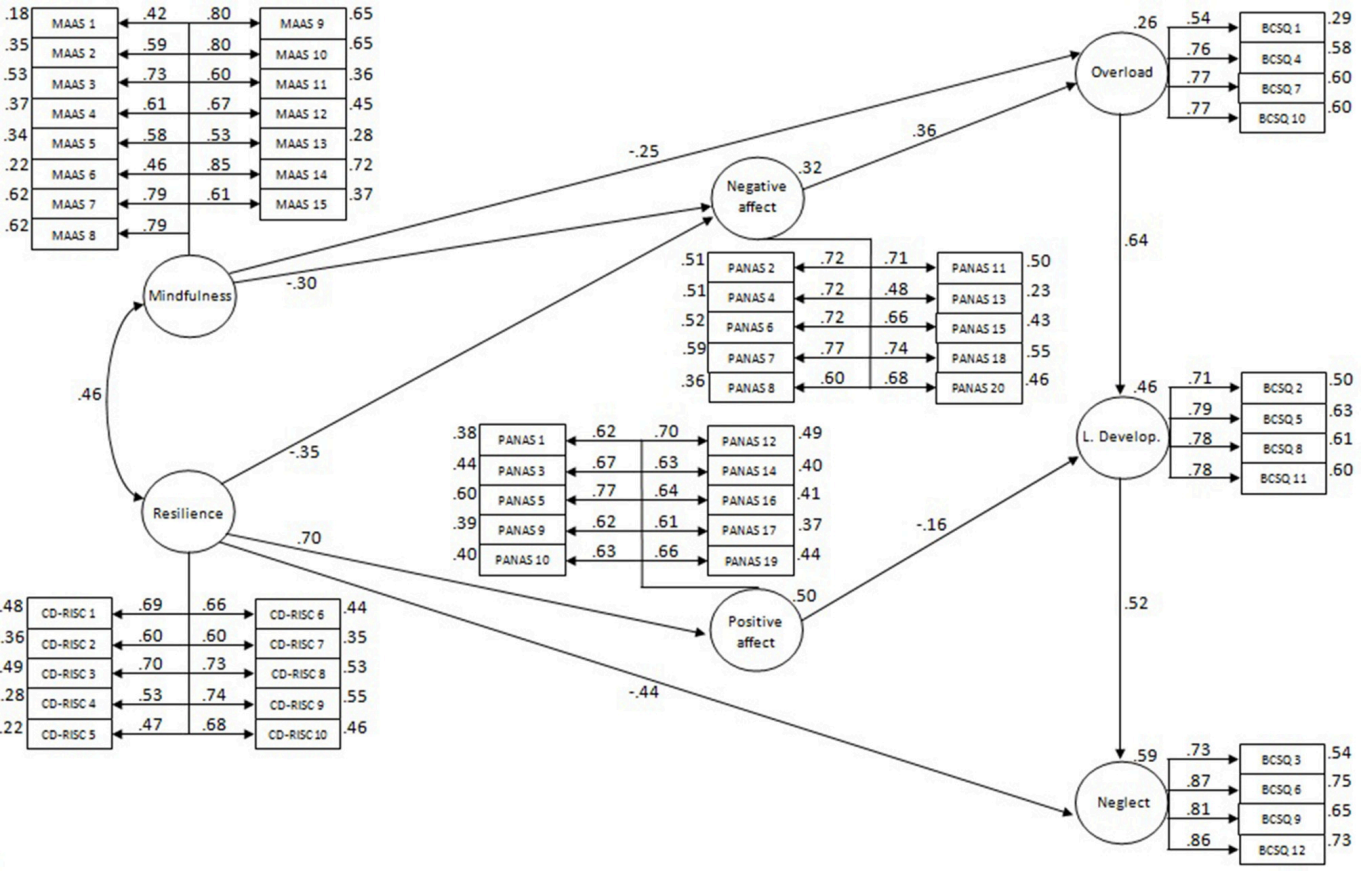
**Pattern of associations among mindfulness and resilience with the burnout types taken into account affects as mediators**. Standardized estimates from SEM. Circles represent latent constructs and rectangles are observable variables. Factor weights are over the arrows and the percentage of explained variance over the circles and boxes.

The fit of the saturated hypothetical model was very good (GFI = 0.96; AGFI = 0.96; RMSR = 0.06; NFI = 0.95; RFI = 0.95; PRATIO = 0.95). However, in order to improve parsimony, the following links with very small effects were removed: mindfulness to lack of development (γ = −0.03), mindfulness to positive affect (γ = 0.03), mindfulness to neglect (γ = −0.09), negative affect to lack of development (β = 0.02), negative affect to neglect (β = −0.03), resilience to overload (γ = 0.02), resilience to lack of development (γ = 0.09), positive affect to overload (β = −0.08), and positive affect to neglect (γ = −0.09). With the removal of these links, the same fit values were shown as those using the hypothetical model, but gave a better result for parsimony (PRATIO = 0.96). The residual errors for the endogenous latent variables in terms of disturbance were: positive affect **ζ** = 0.09, negative affect **ζ** = 0.26, overload **ζ** = 0.56, lack of development **ζ** = 0.62, neglect **ζ** = 0.27. The standardized parameters of the selected final parsimonious model are shown in Figure 2.

## Discussion

This is the first study that has examined a potential model of relationships between mindfulness and resilience, and the burnout subtypes, taking into account affects such as intermediary latent factors, in primary care physicians. However, this model might also be used for the other primary care providers (nurses, social workers, or other health personnel), who work in the same context. Other works have indicated the possible relevance of mindfulness, resilience, and affect to burnout (David and Quintão, 2012; Fortney et al., 2013; Arrogante, 2014), but they did not study their specific and combined influence with regard to the different burnout subtypes. A SEM analysis was computed to assess the theory-driven hypotheses related to potential causal paths between constructs, aimed at potentially informing the development of tailored early intervention approaches. Results showed important links between the considered constructs, pointing out different ways for intervention.

An important strength of the present study is the large size of the sample recruited, which allowed us to perform SEM with an adequate ratio according to the model complexity (Kline, 1998). Moreover, generalizability was enhanced because it was carried out using a burnout high risk profession (Al-Sareai et al., 2013). This sample was recruited from all the Spanish regions; however, the participants may not have been representative of the broader population of Spanish primary care doctors, given that all of them were selected because of their participation in an on-line course (it is possible we obtained more motivated professionals because they were actively seeking treatment). Furthermore, by using an online procedure, the potential for errors in the data transcription process was reduced, and the analysis method respected the true non-linear nature of the variables. The main limitation of this study is the fact that the cross-sectional design used did not allow us to draw firm conclusions about the etiology of burnout subtypes. This sort of design only allows for the evaluation of relationships between variables at one point in time, and thus can only suggest possible causal pathways (MacCallum and Austin, 2000). Although a reversed explanatory path could have been possible, and statistical data were similar in terms of fit, we defend the established hypothesis for the following reason: Mindfuness is considered a stable trait associated with neuroimaging findings (Lu et al., 2014; Wang et al., 2014) and other biological variables (Tomfohr et al., 2015), which protects from vulnerability to other psychopathological disorders, such as depression (Paul et al., 2013). Regardless, the cross-sectional design of the study did not allow this hypothesis to be fully confirmed, and it should therefore be considered a limitation.

We observed that the age of participants was around 50, and most were married women living in their own homes with their partner. Almost all were working at that moment and the majority were employed on a permanent contract in the public sector. Almost none practiced mindfulness before the beginning of the course. The multivariate distribution of the items recommended calculating polychoric correlations for further analysis, and the measurement models used were adequate.

The hypothetical structural model showed very good fit to the data; moreover, the removal of the very small slopes maintained the indices in the same values, and even increased parsimony somewhat. In this regard, the parsimonious model explained a high percentage of variance in latent factors and showed an interesting pattern of relationships. First, a moderately high correlation between mindfulness and resilience was observed, which means that both constructs appear associated, as we established above, although maintaining sufficient discriminant validity so as to be treated differently. It has been said that mindfulness may promote resilience and protect against burnout (Olson et al., 2015), perhaps by moderating the impact of work-related stressors through the reduction of sympathetic activation, improving emotion regulation (potentially mediated by the decrease in negative affect), and enhancing coping with psychological challenges (Hölzel et al., 2013; Duchemin et al., 2015; Westphal et al., 2015). This could explain why we observed negative and moderate associations between mindfulness and overload, a first step in the developmental course of burnout. On the other hand, negative and moderately high associations between resilience and neglect were found; in other words, resilience could be related to high levels of engagement in the sense of the willingness to invest one's effort and persistence (potentially meditated by the improvement of positive affect; Jeve et al., 2015).

Negative and moderate associations were found for mindfulness and resilience with negative affect, as have been found in other studies (Glück and Maercker, 2011; Leontjevas et al., 2014). Moreover, negative affect was positively and moderately related to overload, which is consistent with the finding of venting of negative emotions as the main coping strategy used by overloaded workers (Montero-Marín et al., 2014b). On the other hand, resilience was positively and highly related to positive affect, and positive affect was related in a negative and moderately low way to lack of development. This result may reflect the sense of significance and pride, and challenge underlying engagement (Jeve et al., 2015), which may be associated with resilience through increasing positive affect. In general, affects explain a substantial portion of the variance in well-being (Menk Otto et al., 2010). These results suggest that we may be able to modify affects by implementing programs focused on the improvement of mindfulness and resilience.

In summary, interventions based on mindfulness and resilience may exert direct influences on burnout levels, but also indirect ones, through the change in levels of negative and positive affect. In general, mindfulness and resilience could constitute a good tandem against burnout (Olson et al., 2015). The emphasis required for one or another module would depend on the type of distress and burnout experienced. As we have seen, the links between the burnout types reached high and positive values from overload to lack of development, and from lack of development to neglect. Therefore, each type of burnout might be associated with different stages in terms of the developmental course of the syndrome, routed from highest to lowest level of dedication, as has been proposed in other studies (Montero-Marín et al., 2009, 2014a,b). For these reasons, and given our results, mindfulness appears to be a better intervention for preventing first phases of burnout, by reducing negative arousal states, and thereby perceived overload. In contrast, resilience seems to be a good program for treating burnout when the syndrome is in its advanced stages, by rescuing positive affect and leading to balanced involvement, personal development, and engagement.

Primary care physicians are among the specialists who most often have to deal professionally with work-related health problems such as burnout (Regamey et al., 2013). Paradoxically, they also report high levels of this syndrome (Krasner et al., 2009). Burnout in these professionals must be prevented and treated in order to prevent their personal consequences and also repercussions for patients. The results of this cross-sectional, individual differences study initially suggest that interventions focused on improving mindfulness and resilience may be helpful in the prevention and treatment of burnout, at least in the specific population of primary care physicians.

## Funding

This study was supported by the Research Network on Preventative Activities and Health Promotion (RD06/0018/0017) and the Aragon Health Sciences Institute. MT was supported by a Consolidator Grant of the European Research Council (ERC-2011-StG 20101124) awarded to Sander Koole.

### Conflict of interest statement

The authors declare that the research was conducted in the absence of any commercial or financial relationships that could be construed as a potential conflict of interest.
